# Eye movements and ERP biomarkers for face processing problems in avoidant attachment-style individuals

**DOI:** 10.3389/fnbeh.2023.1135909

**Published:** 2023-05-19

**Authors:** Simeng Gu, Yao Jiang, Mei Liu, Yumeng Li, Yuan Liang, Rou Feng, Minghong Xu, Fushun Wang, Jason H. Huang

**Affiliations:** ^1^Department of Psychology, School of Medicine, Jiangsu University, Zhenjiang, Jiangsu, China; ^2^Institute of Brain and Psychological Science, Sichuan Normal University, Chengdu, Sichuan, China; ^3^Department of Neurology, Lianyungang Hospital of Chinese Medicine, Affiliated Hospital of Nanjing University of Chinese Medicine, Nanjing, China; ^4^Department of Neurosurgery, Baylor Scott & White Health Center, Temple, TX, United States; ^5^Department of Surgery, Texas A&M University, Temple, TX, United States

**Keywords:** avoidant attachment, eye-tracking, ERP, facial expressions, deactivating strategies, MDD

## Abstract

**Background:**

Avoidant attachment poses a serious risk to intimate relationships and offspring. However, there are few studies on the face-processing characteristics and impairments of avoidant individuals based on basic emotion theory. Therefore, this study investigated the issues of emotional processing and deactivation strategies in individuals with avoidant attachment.

**Methods:**

Avoidant and secure individuals were recruited to participate in an eye-tracking experiment and a two-choice oddball task in which they had to distinguish facial expressions of basic emotions (sadness, anger, fear, disgust, and neutral). Eye fixation durations to various parts of the face, including the eyes, nose, and mouth, were measured, and three event-related potentials (ERP) components (P100, N170, and P300) were monitored.

**Results:**

Avoidant individuals could not process facial expressions as easily as secure individuals. Avoidant individuals focused less on the eyes of angry faces when compared to secure individuals. They also exhibited a more positive P100 component and a less negative N170 component when processing faces and a larger amplitude of the P300 component than secure individuals when processing emotional expressions.

**Conclusion:**

Avoidant individuals use deactivating strategies and exhibit specific characteristics at different stages, which are of great significance in social interaction.

## Highlights

- Avoidant individuals have problems with emotional face recognition and cannot process information related to the whole face.
- Avoidant individuals focus less on angry eyes than secure individuals.
- The vigilance-avoidance theory may be evident in the early stages of face processing.
- Avoidant individuals show a more positive P300 component at the late stage of face processing.

## 1. Introduction

Attachment theory focuses on how the quality of one’s interactions with their caregivers during childhood influences their behavior and emotional regulation, in addition to how a person perceives and processes emotional content later in life, such as recognizing the facial expressions of others (Suslow et al., 2009; Liu et al., 2017; Kungl et al., 2022; Momeni et al., 2022). Infants exhibit an attentional bias for faces over patterns or objects and can distinguish between positive and negative faces even when they are only a few months old (Leppänen, 2016; Pyykkö et al., 2019). Also, infants who have received insufficient attention and negative responses from caregivers may be influenced by these memories and become accustomed to deactivating strategies when responding to emotional information later in life (Ainsworth et al., 1978; Braungart-Rieker et al., 2019; Liu et al., 2020). Perceptual processing of emotional stimuli and preferences for emotional regulation vary across individuals with different attachment styles. Secure individuals are assumed to freely evaluate emotional information using balanced responses (Dewitte et al., 2007). In contrast, avoidant individuals are accustomed to avoiding activation of the attachment system; they remain deactivated in an attempt to maximize their distance from attachment figures and to limit proximity-related concerns or painful memories (Shaver and Mikulincer, 2002; Liu et al., 2017; Mikulincer and Shaver, 2019).

Recent advances in basic emotion theory (BET) have improved our understanding of emotional processing (He et al., 2021). According to this theory, a limited number of basic emotions can be modulated by distinct neurophysiological mechanisms and expressed through specific cross-cultural facial expressions (Gu et al., 2019a,b, 2022). For example, the processing of emotional stimuli containing information that influences interpersonal communication, such as fear and anger, may be affected at different levels (Liang et al., 2021a,b). However, when investigating the effects of avoidant attachment on the processing of emotional faces, previous studies usually only included angry, neutral, or happy faces (Dan and Raz, 2012; Irak et al., 2020), ignoring the differences in the processing of different threatening stimuli or negative emotional faces (e.g., sadness, anger, fear, and disgust) across attachment types. Therefore, it is necessary to systematically study the impact of avoidant attachment on the processing of facial expressions of different basic emotions, particularly negative emotions.

Avoidant individuals employ more complex and elaborate preemptive and postemptive strategies to regulate emotional processing at different stages (Huang et al., 2019). At the initial stage of encoding the structural properties of emotional stimuli, avoidant individuals tend to minimize the attended information to suppress the activation of the attachment system (Fraley and Brumbaugh, 2007; Zheng et al., 2015). Avoidant individuals then adopt postemptive strategies to avoid further processing of encoded information, such as encoded thoughts or memories. Thus, avoidant individuals’ strategies for coping with the emotional information they receive are affected by the processing stages.

Moreover, recordings of brain responses using electroencephalography (EEG) would provide more valuable evidence of secondary strategies for the perceptual and attentional processing of emotional information, owing to their higher temporal sensitivity (Eimer and Holmes, 2002; Peltola et al., 2020). Avoidant individuals adopt preemptive strategies to orient their attention away from negative expressions (Dewitte and Houwer, 2008; Dewitte, 2011) and show a less negative N2 component, indicating that they restrict their attention to encoding the structural information of faces (Zhang et al., 2008). However, some alternative findings have shown that avoidant individuals appear to be strongly engaged in perceptual vigilance for emotional stimuli, with emotional faces being recognized faster than neutral ones and the amplitudes of C1 and P1 being larger for angry faces (Dan and Raz, 2012; Zheng et al., 2015). In addition, they exhibit an enhanced amplitude for the N170 component in response to angry faces when compared to secure individuals (Irak et al., 2020). These results indicate a perceptual bias, especially in the initial stages, and are consistent with the vigilance-avoidance theory, which suggests that avoidant individuals exhibit initial vigilance to threats followed by disengagement and attentional avoidance (Derakshan et al., 2007; Myers, 2010). This may encourage avoidant individuals to quickly identify potential sources of threat and prepare for the use of postemptive strategies in later processing.

The phenomenon of postemptive strategies being applied by avoidant individuals when processing facial expressions has been discussed in the extant literature. The P300 component is often used to index cognitive processing in the brain, such as attention, concentration, and mental and emotional processing (Casali et al., 2016; Zhong et al., 2019). Ma et al. (2017) found that avoidant nulliparous women showed smaller P300 amplitudes for all infant faces than secure women, suggesting that they may have adopted postemptive strategies to avoid processing infant faces. However, Zilber et al. (2007) did not find any differences between the P300s of avoidant and secure individuals. Therefore, it is unclear how avoidant individuals react to facial expressions and what the different processing modes are at each stage.

To investigate how avoidant and secure adults process different facial expressions, including angry, fearful, disgusting, sad, and neutral faces, we conducted an eye-tracking experiment in Experiment 1. Our hypothesis was that, compared to secure individuals, avoidant subjects would have shorter fixation durations on negative faces than secure individuals. To provide further evidence regarding the perceptual and attentional processing of emotional information, in Experiment 2, we also recorded brain-related responses using electroencephalography (EEG), which has a higher temporal sensitivity (Peltola et al., 2020). Specifically, in Experiment 2, we investigated the deactivation strategies of avoidant individuals, particularly their responses to facial expressions during different processing stages. We expected avoidant individuals to adopt preemptive strategies during the initial encoding of facial expressions (indicated by the larger P100 and N170) and postemptive strategies during the further processing of the encoded emotional stimuli (indicated by the smaller P300).

## 2. Experiment 1

### 2.1. Materials and methods

#### 2.1.1. Participants

We posted the Experiences in Close Relationships Inventory (ECR) scale and received 500 answers. We selected 34 women (*M* = 19.84, *SD* = 1.25) who scored high on the avoidant scale (above the top 27%) but low on the anxious scale (below the bottom 27%) for the avoidant group, and another 33 participants (*M* = 20.72, *SD* = 1.86) who scored low on both scales (below the bottom 27%) for the secure group. After excluding two participants in each group due to low response accuracy or unusable eye-tracking data, 30 avoidant women and 30 secure women remained. All participants were healthy, right-handed undergraduate students at Sichuan Normal University in Chengdu, China. In addition, they all signed an informed consent form before the experiment. All the experimental procedures for this study were conducted in strict accordance with the institutional guidelines of the Ethics Committee of Sichuan Normal University (Chengdu, China).

All participants were asked to complete the State-Trait Anxiety Inventory (STAI) questionnaire to determine their level of anxiety before participating in the experiment (Spielberger, 1972; Sinclair et al., 2019). The result of the *t*-test showed that there was no difference between the avoidant group (*M* = 39.133, *SD* = 8.557) and the secure group (*M* = 43.467, *SD* = 8.565), with *p* > 0.05.

#### 2.1.2. Experiences in close relationships inventory

Participants were asked to complete the Chinese Volume of Experiences in Close Relationships Inventory (ECR-C) to assess adult attachment styles (Ainsworth et al., 1978; Bowlby, 1982). The ECR scale was originally developed by Brennan et al. (1998) and later used in China after being revised by Li and Kato (2006). The self-report questionnaire contained 36 items on the anxiety and avoidance dimensions. The anxiety scale (18 items; alpha = 0.85) assessed participants’ concerns about abandonment and departure (e.g., “I worry about being abandoned”), whereas the avoidance scale (18 items; alpha = 0.87) reflected the degree of intimacy and independence (e.g., “I become nervous when partners get too close to me”). Each item was rated on a seven-point Likert scale ranging from 1 (“not at all”) to 7 (“very much”).

#### 2.1.3. Procedure

**FIGURE 1:**
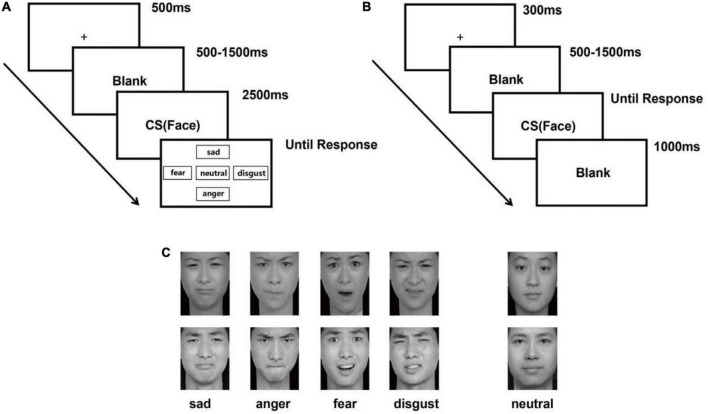
Experimental procedure. **(A)** Task for Experiment 1. Participants were asked to click a button using the mouse to describe the emotion shown on the screen: “sadness” (up), “disgust” (right), “anger” (down), “fear” (left), or “neutral” (center) (Wang and Luo, 2005; Gong et al., 2011). **(B)** Task for Experiment 2. Participants were asked to respond to facial expressions by pressing “f” (negative) and “j” (neutral). **(C)** Examples of the emotional faces (sadness, anger, fear, and disgust) and neutral faces presented in both experiments.

A visualization task was employed in Experiment 1. This task included 80 emotional faces (20 sad, 20 angry, 20 fearful, and 20 disgusted faces) and 20 neutral faces selected from the Chinese Facial Affective Pictures System (CFAPS) (260 × 300 pixels). Each face was presented once (Wang and Luo, 2005). There were four blocks of 30 trials each. In each trial, a fixation cross was presented for 500 ms, followed by a variable inter-trial interval between 500 ms and 1,500 ms to avoid stimulus onset anticipation. Then, a picture of emotional or neutral faces was presented in the center, which lasted for 2,500 ms. Participants were asked to look at the faces and use the monitor to choose the type of emotion after the faces disappeared. As soon as the participants selected an emotion, the selection screen disappeared, and the next trial began. The details of this trial are presented in Figures 1A, C.

#### 2.1.4. Eye-tracking recording

Fixation durations were recorded binocularly at 1,000 Hz using the Eyelink 1000 plus desktop mount (SR Research Ltd., ON, Canada). Calibration and validation were performed before each block to ensure high efficiency. For the calibration, the participants were asked to track nine random points on the screen, and then the eye tracker was adjusted until the average tracking error of the visual angle was less than 0.4%. The same random points were also used to measure the differences between the eye fixation position and the target fixation position in the validation procedure.

### 2.2. Analysis

Fixation measures were calculated using the Eyelink DataViewer and analyzed using SPSS 23. Three areas of interest (AOIs) were defined for each face during the separation segments: an Eye AOI, a Nose AOI, and a Mouth AOI. The duration of fixation on the AOIs was chosen as an index. All data from the correct response trials were analyzed. All data were analyzed using repeated measures analysis of variance (ANOVA) with Bonferroni correction for multiple comparisons. In addition, effect sizes were presented as partial eta-squared (η^2^_*p*_) to indicate effects (Cohen, 1988).

### 2.3. Results

#### 2.3.1. Behavioral data

Response time and accuracy were analyzed using a 2 (attachment type: avoidant or secure) × 5 (emotional face: sad, angry, fearful, disgusted, or neutral) repeated measures ANOVA.

**FIGURE 2:**
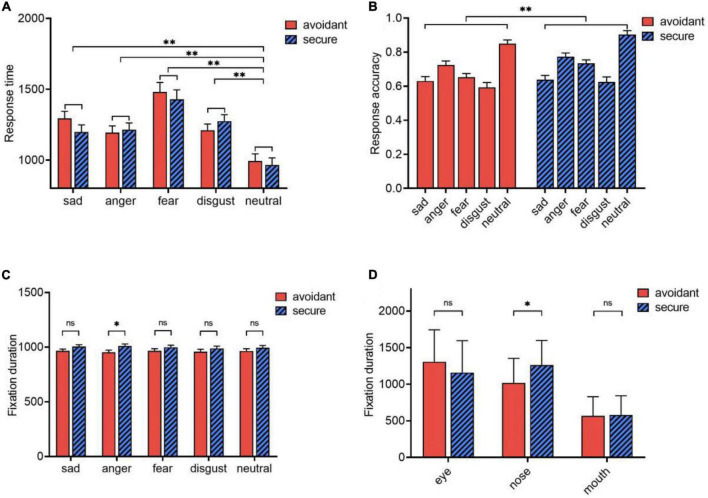
Eye movements. **(A)** Response time of avoidant individuals and secure individuals; **(B)** Response accuracy of avoidant individuals and secure individuals; **(C)** Effects of attachment types and emotional faces on fixation duration; **(D)** Effects of attachment types and AOIs on fixation duration. *p < 0.05; **p < 0.001; ns, no significance.

For response time, the main effect of emotional faces was significant [*F*(4,55) = 22.865, *p* < 0.001, η^2^_*p*_ = 0.624]. The response time for neutral faces (*M* = 979.082, *SE* = 35.640) was significantly shorter than for angry (*M* = 1204.317, *SE* = 33.558), sad (*M* = 1245.857, *SE* = 35.669), disgusted (*M* = 1242.356, *SE* = 31.826), and fearful (*M* = 1454.120, *SE* = 47.535, *p* < 0.001) faces (Figure 2A). However, there were no significant differences between attachment types (*p* = 0.742), or between the interaction effect of emotion and attachment types (*p* = 0.172).

A significant main effect of attachment type was observed for response accuracy [*F*(1,5860) = 7.903, *p* = 0.007, η^2^_*p*_ = 0.120]. Avoidant individuals (*M* = 0.690, *SE* = 0.011) were less accurate in their responses than secure individuals (*M* = 0.7350, *SE* = 0.011; Cohen’s *d* = −0.076) (Figure 2B). Additionally, the main effect of emotional faces was significant [*F*(4,55) = 63.309, *p* < 0.001, η^2^_*p*_ = 0.822]. Specifically, the response accuracy for neutral faces (*M* = 0.877, *SE* = 0.015) was higher than that for angry faces (*M* = 0.749, *SE* = 0.016), fearful (*M* = 0.694, *SE* = 0.015), sad (*M* = 0.634, *SE* = 0.018), and disgusted faces (*M* = 0.608, *SE* = 0.015); and the differences in response accuracy for angry (*M* = 0.749, *SE* = 0.016), fearful (*M* = 0.694, *SE* = 0.015), and disgusted faces (*M* = 0.608, *SE* = 0.015) were independently significant (*p* < 0.001). However, there was no significant interaction between emotion and attachment type (*p* = 0.550).

#### 2.3.2. Eye movements

Fixation durations were analyzed using a 2 (attachment type: avoidant or secure) × 5 (emotional face: sad, angry, fearful, disgusted, or neutral) × 3 (AOI: eye, nose, or mouth) ANOVA. The attachment type × emotion interaction for fixation duration was significant [*F*(4,55) = 3.788, *p* = 0.012, η^2^_*p*_ = 0.216]. Avoidant individuals (*M* = 952.964, *SE* = 19.846) fixated for shorter periods than secure individuals on angry face trials (*M* = 1010.232, *SE* = 19.846; *p* = 0.046, Cohen’s *d* = −0.5358). However, there were no differences between individuals with the two attachment types in trials with other emotional faces (*p* > 0.05) (Figure 2C).

The attachment type × AOI interaction for fixation duration was also significant [*F*(2,57) = 4.639, *p* = 0.014, η^2^_*p*_ = 0.14]. For the nose, the fixation duration of avoidant subjects (*M* = 1014.472, *SE* = 61.252) was significantly shorter than that of secure individuals (*M* = 1261.918, *SE* = 61.252; *p* = 0.006, Cohen’s *d* = −0.7502). However, for the mouth and eyes, the fixation duration of avoidant respondents and secure individuals did not differ significantly (*p* = 0.838, *p* = 0.195) (Figure 2D).

In addition, the interaction of emotional face × AOI was significant. However, the main effect of the attachment type was not significant (*p* = 0.169). The fixation duration of the avoidant individuals (*M* = 961.768, *SE* = 19.221) was slightly shorter than that of the secure individuals (*M* = 999.655, *SE* = 19.221). Moreover, the interaction between emotion, AOI, and attachment type was not significant (*p* = 0.896), nor was the main effect of emotion (*p* = 0.485).

## 3. Experiment 2

### 3.1. Materials and methods

#### 3.1.1. Participants

A total of 20 female college students were selected for the avoidant group (*M* = 19.83, *SD* = 0.69) and twenty for the secure group (*M* = 21.06, *SD* = 2.32), based on the same criteria as in Experiment 1. None of the selected students had participated in our previous eye-tracking experiment. Two secure women and two avoidant women were excluded from the sample because of excessive artifacts, leaving 18 subjects in each group. As in Experiment 1, all participants were asked to complete an STAI questionnaire to assess their level of anxiety before participating in the experiment. The results of the *t*-test showed that there was no difference between the avoidant group (*M* = 38.941, SD = 9.351) and the secure group (*M* = 43.588, SD = 9.440), *p* > 0.1.

#### 3.1.2. Experiences in close relationships inventory

The alpha values of internal consistency in the ERP experiment were 0.872 for the avoidance scale and 0.848 for the anxiety scale.

#### 3.1.3. Procedure

A two-choice oddball paradigm was used in Experiment 2. A total of 24 facial expressions (6 sad, 6 angry, 6 fearful, and 6 disgusted) and 6 neutral faces were selected from the CFAPS (260 × 300 pixels) (Wang and Luo, 2005). According to the paradigm, the neutral face was presented as a normal stimulus 68 times, and all negative faces were presented separately as target stimuli 8 times in each block; there were six blocks in total. On each trial, a fixation cross was presented for 300 ms, followed by a variable inter-trial interval between 500 ms and 1,500 ms to avoid stimulus onset anticipation; a picture of the emotional or neutral face was shown until the response. Participants were instructed to press “f” when they saw a negative face and “j” when they saw a neutral one. After the response, the face would disappear, and the next trial would be shown after 1,000 ms of blank screen time. The specific procedure for this trial is presented in Figure 1B.

#### 3.1.4. EEG data collection

Continuous EEG data were recorded in 64 channels using a Brain-Vision Recorder 2.1 (Brain Products GmbH, Munich, Germany) arranged in the international standard 10/10 system with 64 Ag/AgCl active electrodes and an actiCHamp amplifier. The recording impedance of all channels was less than 5 kΩ and the sampling rate of the offline recording was 500 Hz. The electrical signals were amplified with a 0.01–100 Hz bandpass filter. All channels were referenced to the FCz during recording. The vertical electrooculogram (EOG) was recorded by replacing the electrode below the right eye, and the horizontal EOG was recorded using an offline FT9.

### 3.2. Analysis

Electroencephalography data were processed using MATLAB 2018b (MathWorks, USA) and the EEGLAB toolbox (Delorme and Makeig, 2004) and further analyzed using SPSS 23. After using the BESA file to determine the channel location, all channels were re-referenced offline to the mean activity of the bilateral mastoids. All EEG data were separately filtered with a high-pass filter (0.1 Hz) and a low-pass filter (30 Hz) to remove noise. Subsequently, the EOG was extracted, and ICA was applied to remove artifacts such as EOG and myoelectricity. After completing the baseline correction, the remaining artifacts of continuous data with amplitudes exceeding ± 100 μV were automatically eliminated before segmentation. EEG data without artifacts were epoched 800 ms after picture onset and computed with an additional 200 ms pre-stimulus baseline. Trials with correct responses were averaged for each condition.

**FIGURE 3:**
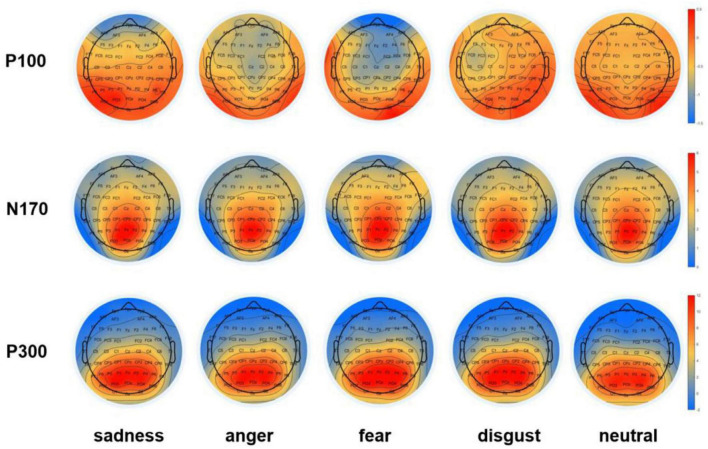
Topographic maps of ERP components. Topographic maps of the occipital P100, the lateral occipital N170, and the parietal P300 components in negative (sadness, anger, fear, and disgust) and neutral conditions during the time windows of 50–100 ms (P100), 160–210 ms (N170), and 300–500 ms (P300).

The ERP components in three-time windows previously thought to be sensitive to adult attachment and facial expression processing, namely, P100, N170, and P300, were analyzed. These were named and quantified as the most negative or positive ERP activity obtained at different time intervals after the stimulus onset, namely the occipital P100 (50–100 ms; O1/2) (Felmingham et al., 2003; Herrmann et al., 2005; Boutsen et al., 2006; Waller et al., 2015; Irak et al., 2020); the lateral-occipital N170 (160–210 ms; P7/8, PO7/8) (Zheng et al., 2015; Ma et al., 2017; Irak et al., 2020); and the parietal P300 (300–500 ms; P3/4, P7/8, PO7/8) (Ma et al., 2017; Figure 3). For each component, the mean amplitudes at selected locations in the time window were analyzed independently. All data were analyzed using repeated measures ANOVA, with Bonferroni correction for multiple comparisons. Moreover, effect sizes were presented as partial eta-squared (η^2^_*p*_) to indicate the effects (Cohen, 1988).

### 3.3. Results

#### 3.3.1. Behavioral data

**FIGURE 4:**
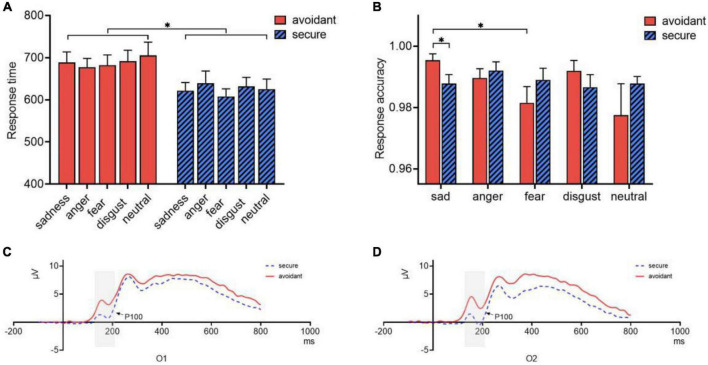
Characteristics of ERP in different attachments. **(A)** Response time of avoidant and secure individuals; **(B)** Response accuracy of avoidant and secure individuals; **(C)** P100 components of avoidant and secure individuals at O1; **(D)** P100 components of avoidant and secure individuals at O2. *p < 0.05.

Response time and accuracy were analyzed using a 2 (attachment type: avoidant or secure) × 5 (emotional faces: sad, angry, fearful, disgusted, or neutral) ANOVA. The main effect of attachment type on response time was significant [*F*(1,34) = 6.53, *p* = 0.015, η^2^_*p*_ = 0.161]. A *post hoc* analysis showed that the response time of secure individuals (*M* = 625.03, *SE* = 17.677) was shorter than that of avoidant individuals (*M* = 688.92, *SE* = 17.677, Cohen’s *d* = 0.877) (Figure 4A). However, there was no significant main effect of emotional faces (*p* = 0.70) or interaction effect (*p* = 0.648).

Regarding response accuracy, the interaction between attachment type and emotional faces was significant [*F*(4,31) = 3.061, *p* = 0.031, η^2^_*p*_ = 0.283]. Accuracy for sad faces (*M* = 0.995, *SE* = 0.003) was significantly higher than that of fearful faces (*M* = 0.981, *SD* = 0.005) in avoidant individuals (*p* = 0.015, Cohen’s *d* = 0.412), but not significant in secure individuals (*p* = 1.00). In the sad face trial, the accuracy of avoidant individuals (*M* = 0.995, *SE* = 0.003) was significantly higher than that of secure individuals (*M* = 0.988, *SE* = 0.003, *p* = 0.02, Cohen’s *d* = 0.566) (Figure 4B). However, the main effects of emotional faces (*p* = 0.168), and attachment type (*p* = 0.65) were not significant.

#### 3.3.2. P100

A mixed 2 (attachment type: avoidant or secure) × 5 (emotional face: sad, angry, fearful, disgusted, or neutral) × 2 (hemisphere: left or right) ANOVA for the P100 component indicated significant main effects of attachment type [*F*(1,32) = 5.831, *p* = 0.022, η^2^_*p*_ = 0.154]. Avoidant individuals showed larger P100 component amplitudes than secure individuals. Moreover, the interaction between attachment type and hemisphere was significant [*F*(1,32) = 6.959, *p* = 0.013, η^2^_*p*_ = 0.179]. Multiple comparisons showed that avoidant individuals (*M* = 0.926, *SE* = 0.181) showed larger P100 components than secure individuals (*M* = 0.204, *SE* = 0.181) in the right hemisphere (*p* = 0.008, Cohen’s *d* = 3.989). However, there was no difference in the left hemisphere (*p* = 0.114) (Figures 4C, D).

#### 3.3.3. N170

A mixed 2 (attachment type: avoidant or secure) × 5 (emotional face: sad, angry, fearful, disgusted or neutral) × 2 (hemisphere: left or right) ANOVA analysis for the P100 component indicated a significant main effect of attachment type [*F*(1,32) = 5.477, *p* = 0.026, η^2^_*p*_ = 0.146] and hemisphere [*F*(1,32) = 5.350, *p* = 0.027, η^2^_*p*_ = 0.143] and a significant interaction between emotion and hemisphere [*F*(4,29) = 2.787, *p* = 0.045, η^2^_*p*_ = 0.278].

**FIGURE 5:**
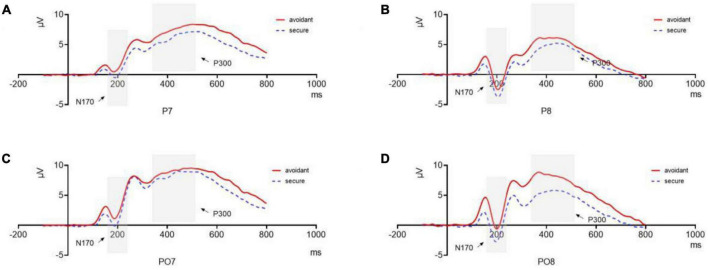
N170 and P300 components. **(A)** N170 and P300 components of avoidant and secure individuals at P7; **(B)** N170 and P300 components of avoidant and secure individuals at P8; **(C)** N170 and P300 components of avoidant and secure individuals at PO7; **(D)** N170 and P300 components of avoidant and secure individuals at PO8.

Secure individuals (*M* = −0.958, *SE* = 0.659) showed a more negative N170 component compared to avoidant individuals (*M* = 1.224, *SE* = 0.659, Cohen’s *d* = 3.311). They exhibited a more negative N170 component in the right hemisphere (*M* = −0.479, *SE* = 0.463) than that in the left hemisphere (*M* = 0.745, *SE* = 0.600, Cohen’s *d* = 2.284). A *post hoc* analysis indicated that the amplitude of the N170 component in the right hemisphere was significantly larger than that in the left hemisphere during sad processing (*M*_*R*_ = −0.683, *SE*_*R*_ = 0.586; *M_*L*_* = 0.829, *SE_*L*_* = 0.651, Cohen’s *d* = 2.441), angry (*M*_*R*_ = −0.364, *SE_*R*_* = 0.479; *M*_*L*_ = 1.089, *SE*_*L*_ = 0.624, Cohen’s *d* = 2.612), and disgusted (*M*_*R*_ = −0.783, *SE*_*R*_ = 0.513; *M_*L*_* = 0.641, *SE*_*L*_ = 0.627, Cohen’s *d* = 2.486) faces. However, there were no differences between trials with fearful and neutral faces (*p* > 0.1) (Figures 5A, B).

#### 3.3.4. P300

A mixed 2 (attachment type: avoidant or secure) × 5 (emotional face: sad, angry, fearful, disgusted, or neutral) × 6 (hemisphere: left or right) ANOVA for the P300 component indicated significant main effects of attachment type [*F*(1,32) = 5.093, *p* = 0.031, η^2^_*p*_ = 0.137] and hemisphere [*F*(1,32) = 5.466, *p* = 0.026, η^2^_*p*_ = 0.146]. Avoidant individuals (*M* = 8.851, *SE* = 0.680) showed an increased amplitude of the P300 component compared to secure individuals (*M* = 6.681, *SE* = 0.680, Cohen’s *d* = 3.191). Moreover, the P300 component was larger in the left hemisphere (*M* = 8.368, *SE* = 0.585) than in the right one (*M* = 7.164, *SE* = 0.502, Cohen’s *d* = 2.209).

The interaction between hemisphere and emotion was also significant [*F*(4,29) = 2.836, *p* = 0.042, η^2^_*p*_ = 0.281]. The amplitudes of the P300 component in the left hemisphere were significantly larger than those in the right hemisphere during sad processing (*M*_*L*_ = 8.362, *SE*_*L*_ = 0.732; *M_*R*_* = 6.767, *SE_*R*_* = 0.698, *p* = 0.010, Cohen’s *d* = 2.230), angry (*M*_*L*_ = 8.359, *SE_*L*_* = 0.594; *M_*R*_* = 6.995, *SE*_*R*_ = 0.465, p = 0.017, Cohen’s *d* = 2.557) and disgusted faces (*M*_*L*_ = 8.592, *SE*_*L*_ = 0.684; *M_*R*_* = 7.218, *SE*_*R*_ = 0.614, *p* = 0.014, Cohen’s *d* = 2.114), but there were no differences between the two hemispheres for fearful trials (*M*_*L*_ = 8.663, *SE*_*L*_ = 0.625; *MR*_*R*_ = 7.568, *SE_*R*_* = 0.581, *p* = 0.057) and neutral faces (*M_*L*_* = 7.866, *SE_*L*_* = 0.519; *MR*_*R*_ = 7.273, *SE_*R*_* = 0.442, *p* = 0.211) (Figures 5C, D).

## 4. Discussion

To the best of our knowledge, this study is the first to use eye-tracking and ERP technologies to examine whether and how attachment styles affect the processing of facial expressions of different basic emotions and the processing strategies of avoidant women from the perspective of basic emotion theory. In the eye-tracking experiment, we found that avoidant individuals exhibited cognitive impairments in facial expression recognition compared to secure individuals. Also, avoidant individuals spent less time looking at the eyes than secure individuals did while processing angry faces. In the ERP experiment, avoidant individuals exhibited a more positive P100 and a larger negative N170 than secure individuals in the early phase. Later, avoidant individuals exhibited a larger P300. Therefore, avoidant individuals have impairments in specific processing characteristics of facial expressions at different stages.

First, we expected that avoidant individuals would not perform as well as secure individuals and would allocate fewer attentional resources when processing facial expressions. Consistent with Stanojeviæ et al.’s (2019) study, avoidant individuals were less accurate in differentiating facial expressions, suggesting a higher threshold for processing negative faces compared to secure individuals (Silva et al., 2015). Previous studies have shown that some patients with major depressive disorder and anxiety have impaired attachment styles (Levitan et al., 2009; Dong et al., 2022; Jiang et al., 2022). In addition, some patients with schizophrenia, autism, or depression score lower on emotional facial expression processing, which may be because they process stimuli differently than the healthy controls (Eisenbarth and Alpers, 2011; Golshani et al., 2021). We then focused on the separate roles of the three AOIs in the decoding of emotional expressions and found that avoidant individuals fixated less on the nose than secure individuals; however, there were no differences in the eyes or the mouth (Juncai and Rong, 2017; Saeedpour-Parizi et al., 2020). Shorter fixation durations indicate lower visual cognitive processing (Wang et al., 2015; Sun and Shi, 2017). Thus, avoidant individuals may be impaired in the processing and decoding of information from the whole face.

In addition, eye-tracking can examine the specific aspects of a stimulus that the individual is either attending to or avoiding (Wieckowski et al., 2019). In our study, avoidant individuals fixated less on angry faces than secure individuals, which suggests inhibition of attentional resources while processing angry faces. Similarly, in a negative affective priming task, attachment avoidance was associated with greater inhibition of both angry and sad faces (Dewitte, 2011). Negative facial expressions, such as angry faces, may remind individuals of negative memories. Moreover, avoidant individuals with high levels of defensiveness show low access to negative memories (Mikulincer and Orbach, 1995; Vandevivere et al., 2014). This attentional bias may also be reflected in the processing strategies used by avoidant individuals.

Our second hypothesis was that avoidant individuals would use preemptive strategies to process initially encoded emotional faces, and postemptive strategies to further process encoded faces. For preemptive strategies in the initial stages, the P100 component was an early visual component that showed sensitivity to the type and intimacy of faces (Balas and Koldewyn, 2013; Earls et al., 2016). When viewing angry faces, avoidant individuals used enhanced automatic attentional resources (P1) to identify negative emotions (Dan and Raz, 2012). Similarly, avoidant individuals recorded higher positive amplitudes of the P100 component than secure individuals while viewing facial expressions of sadness, anger, fear, and disgust, suggesting that avoidant individuals showed a negative bias and were vigilant to negative facial expressions in preparation for later suppression and inhibition.

The N170, a component related to the initial structural encoding of faces, is sensitive to face processing and is moderated by emotional information (Blau et al., 2007; Colombatto and McCarthy, 2017). In our study, avoidant individuals exhibited a less negative N170 component than secure individuals when processing emotional expressions. Using this factor and fixation duration, we demonstrated that avoidant individuals inhibit attention to emotional information and limit cognitive resources, especially in the case of angry or fearful faces. Similarly, Irak et al. (2020) found that avoidant individuals were better at recognizing angry faces and exhibited an increased amplitude of the N170 component compared to positive faces. According to Fraley et al. (2000), avoidant individuals tend to apply preemptive strategies to achieve a defensive function characterized by the restriction of an undesirable event or feeling that may evoke distress and frustration, especially in the early stages of processing (Dan and Raz, 2012). Similarly, Edelstein and Gillath (2008) used an emotional Stroop task to investigate attachment-related differences in emotional processing biases. They also found that avoidant attachment was related to reductions in emotional Stroop interference for attachment-related words (e.g., intimacy, loss). Furthermore, Zhang et al. (2008) found that avoidant individuals were less negative for N2, which reflects the structural encoding of faces after automatic face processing. They also showed less negativity toward the N400 component. Therefore, our results suggest that avoidant individuals restrict their attentional resources to encoding the structural properties of faces, especially when processing angry and fearful faces (Zheng et al., 2015).

Combining the results of the P100 and N170 components, we suspected that the vigilance-avoidance theory might also occur in the early stages of face processing. In a masked affective priming study, avoidant participants showed an early enhanced response to emotional information, followed by cognitive suppression at a controlled processing level (Donges et al., 2015). In addition, Chun et al. (2015) reported a dual process of avoidant attachment, in which avoidant individuals were vigilant to contemptuous faces when they were presented for 100 ms; however, they disengaged from facial stimuli by 750 ms. Similarly, we also found a significant P100 component, indicating that avoidant individuals were initially vigilant to all negative faces in the initial stages. More interestingly, we observed an early avoidant tendency in these individuals at about 200 ms, which suggests that disengagement may occur much earlier. Thus, avoidant individuals tend to adopt preemptive strategies at an early stage while processing emotional faces, which are characterized as being vigilant to threatening stimuli, and then quickly adopt repressive coping strategies.

According to the vigilance-avoidance theory, avoidant individuals exhibit a bias against anger and fear because such negative stimuli activate their earlier negative self-relevant schemas and related autobiographical memories from birth. In avoidant individuals, both fearful and disgusted faces increased the negative amplitude of the N170 component relative to neutral faces at P8, indicating a bias for fearful and disgusted faces over neutral faces in the early stages of processing. Zheng et al. (2015) observed a larger N170 amplitude in highly avoidant individuals while they viewed negative or positive faces than while viewing neutral faces. Therefore, in the initial stage, avoidant individuals exhibit a negative bias to suppress the negative effects of earlier memories.

The second hypothesis was that avoidant individuals would suppress and exhibit a smaller P300 component in the later stages, which was not confirmed in our study (Ma et al., 2017). In contrast, we found an enhanced P300 in avoidant individuals compared to secure individuals. Zilber et al. (2007) also found that highly avoidant individuals had a more positive P300 component than low avoidant individuals. They explained that postemptive strategies should probably be taken at a higher level and should not be reflected in the immediate processing of the incoming information. On the one hand, participants were only required to judge whether the faces were negative, which may not require higher cognitive resources. On the other hand, suppression is also a type of effort that requires cognitive resources (Kerr-Gaffney et al., 2020). Therefore, for avoidant individuals, an elevated P300 may indicate that more cognitive resources were used while processing negative faces.

### 4.1. Limitations and future research directions

Our study has some limitations. First, although repeated measures were used, the relatively small sample size may have reduced the power of the effects and increased the margins of error. Moreover, our sample consisted of all female participants. A meta-analysis revealed gender differences in attachment styles; men showed higher avoidance and lower anxiety than females (Del Giudice, 2011). Future studies can consider the impact of participant gender and increase the sample size. Second, a self-report questionnaire can be used to assess attachment among a large number of participants in a short period. In our study, the ECR is used to assess the two-dimensional measure of attachment styles. Future research could use different instruments, such as the Relationship Questionnaire or the Adult Attachment Interview, to confirm or include a broader range of attachment styles. Third, we applied the same stimuli (unfamiliar emotional faces) but with different paradigms in Experiment 1 and Experiment 2. Some studies have found that attachment-related stimuli may better activate the attachment system and influence the responses of individuals with different attachment styles (Read et al., 2018). Thus, future studies should consider using some attachment-relevant stimuli in different forms and a consistent experimental paradigm to increase the consistency of the results.

## 5. Conclusion

We used eye-tracking and event-related potential (ERP) experiments to investigate the attentional bias and deactivation strategies of avoidant individuals at different stages of facial expression processing based on basic emotion theory. Compared to secure individuals, avoidant individuals showed more impairment in the processing of facial expressions. They used specific processing orders and exhibited a processing bias against angry faces. In addition, in the initial stages, avoidant individuals adopted preemptive strategies to be vigilant and simultaneously suppress the processing of angry and fearful faces. We found a more positive P300 for negative faces in avoidant individuals at a later stage, which may reflect impairments in the processing of encoded faces.

## Data availability statement

The original contributions presented in this study are included in the article/supplementary material, further inquiries can be directed to the corresponding authors.

## Ethics statement

The studies involving human participants were reviewed and approved by the Ethics Committee of Sichuan Normal University (Chengdu, China). The patients/participants provided their written informed consent to participate in this study.

## Author contributions

YJ, RF, SG, and FW designed the study. RF, YJ, ML, and YLi performed the experiments and analyzed the data. SG, YJ, ML, YLia, RF, and FW wrote the manuscript. FW, MX, and JH revised the manuscript. All authors contributed to the article and approved the submitted version.
